# Real-World Wrist-Derived Digital Mobility Outcomes in People with Multiple Long-Term Conditions: A Comparison of Algorithms

**DOI:** 10.3390/bioengineering12101108

**Published:** 2025-10-15

**Authors:** Dimitrios Megaritis, Lisa Alcock, Kirsty Scott, Hugo Hiden, Andrea Cereatti, Ioannis Vogiatzis, Silvia Del Din

**Affiliations:** 1 Faculty of Health and Life Sciences, Northumbria University Newcastle, Newcastle upon Tyne NE1 8ST, UK; ioannis.vogiatzis@northumbria.ac.uk; 2 Translational and Clinical Research Institute, Newcastle University, Newcastle upon Tyne NE4 5PL, UK; lisa.alcock@newcastle.ac.uk (L.A.); kirsty.scott-singer@newcastle.ac.uk (K.S.); silvia.del-din@newcastle.ac.uk (S.D.D.); 3 National Institute for Health and Care Research (NIHR) Newcastle Biomedical Research Centre (BRC), Newcastle University, Newcastle upon Tyne NE4 5PL, UK; 4 School of Computing, Newcastle University, Newcastle upon Tyne NE4 5TG, UK; hugo.hiden@newcastle.ac.uk; 5 Department of Electronics and Telecommunications, Politecnico di Torino, 10129 Turin, Italy; andrea.cereatti@polito.it

**Keywords:** digital mobility assessment, wearable sensors, wrist-worn inertial measurement units (IMUs), real-world monitoring, biomedical signal processing, gait analysis, multimorbidity, algorithm validation, stride length estimation

## Abstract

Digital Mobility outcomes can serve as objective biomarkers of health, but their validation in populations with multiple long-term conditions (MLTCs) based on wrist-worn devices remains unexplored. We refined, improved, and introduced novel algorithms, specifically tailored and adapted for (i) gait sequence detection, (ii) initial contact identification, and (iii) stride length estimation from a single wrist-worn device. Validation was performed using data from 28 participants with co-occurring MLTCs performing a 2.5 h real-world monitoring session. Reference data from an established multi-sensor system were used to assess algorithm performance across diverse gait patterns of co-occurring MLTCs. Twenty-eight participants (mean age 70.4 years, 43% females) had a median of three co-occurring MLTCs. Among six gait sequence detection methods, improved versions of the Kheirkhahan algorithm performed best (accuracy = 0.92, specificity = 0.96). For initial contact detection (nine methods tested), Shin’s algorithm achieved the highest performance index (0.85) followed by McCamley (0.84). Stride length estimation was most accurate using novel approaches based on the Weinberg method (performance index > 0.70). The proposed fine-tuned algorithms, the newly developed adaptive variants, and the foot-length augmented versions demonstrated robust performance, surpassing many existing methods and addressing the complexity of gait patterns in MLTCs. These findings enable scalable, real-time mobility monitoring in complex clinical populations using accessible wearable technology.

## 1. Introduction

Digital Mobility Outcomes (DMOs) have advanced considerably in recent years, emerging as promising tools for the objective assessment of real-world mobility as a reflection of overall health status [1,2,3]. A critical first step toward establishing DMOs as ecologically valid digital biomarkers is the robust demonstration of their algorithmic validity, including accuracy, reliability, and consistency when acquired in real-world settings. Within the scope of the EU-funded Mobilise-D project [4], substantial progress has been made toward this goal, including the development of a comprehensive validation framework, which has been applied across several single long-term conditions (i.e., one primary long-term disease) [1,5]. As such, gait detection algorithms have been developed and validated within the context of single-disease populations, capturing condition-specific gait patterns [1,5]. This disease-specific focus may limit the broader applicability of DMOs as generalizable biomarkers, particularly in real-world settings where multiple long-term conditions (MLTCs) are highly prevalent [6]. Living with MLTCs or ‘multimorbidity’, defined as two or more long-term conditions [7], involves complex, interacting symptoms that cumulatively impair daily function and health-related quality of life (HRQoL) [8]. Given that gait is shaped by the complex interplay of multiple, co-existing long-term conditions, there is a critical need to extend the validation of DMOs to populations with MLTCs, which present with a diverse range of gait impairments, including slow gait, high variability, and asymmetry.

To date, most algorithm development and validation efforts have relied on data captured from a single sensor positioned on the lower back [9]. Although this location provides important biomechanical benefits, ensuring that algorithms perform consistently across additional sensor locations represents a key step toward their integration into commercially available devices and, in particular, their suitability for long-term or large-scale deployment in clinical practice [10]. The wrist position offers a more user-friendly and scalable alternative, being widely adopted through commercially available wearable systems [10,11]. However, this device location poses unique challenges due to the non-fixed orientation of the wrist and the variability of arm movements [12]. These challenges are especially evident in the estimation of outcomes such as stride length, where traditional biomechanical models are inapplicable, thereby requiring the adoption of intensity-based or data-driven methodologies.

To date, large-scale algorithmic ranking validation studies of wrist-worn inertial measurement units (IMUs) have been conducted primarily in single-disease populations and have focused exclusively on gait sequence detection (GSD) [13]. There remains a critical gap in the literature concerning the validation of additional outcomes, such as initial contact detection (ICD), and stride length (SL), in populations with MLTCs (co-occurring long-term conditions), which more accurately represent real-world mobility in clinical populations.

In the present study, we aimed to:

(i) refine, optimize, and introduce novel versions of state-of-the-art algorithms for GSD, ICD, and SL estimation using wrist-worn IMUs;
(ii) technically validate and rank these algorithms in real-world settings.

This validation employed a diverse patient cohort representing five primary long-term conditions with accompanying equally important co-occurring long-term conditions, analyzed collectively to encompass the full spectrum of gait patterns characteristic of multiple long-term conditions in real-world settings.

## 2. Materials and Methods

### 2.1. Study Population

The initial sample included 108 participants from the Mobilise-D Technical Validation Study (TVS) open access dataset [14], each with a primary diagnosis of a single condition: proximal femur fracture (PFF), chronic obstructive pulmonary disease (COPD), Parkinson’s disease (PD), multiple sclerosis (MS), and chronic heart failure (CHF), recruited via their clinical care team [15]. For the present analysis, we included only individuals with MLTCs, so with at least two co-occurring long-term conditions (cardiovascular, metabolic, respiratory, musculoskeletal, or neurological) of equal importance, as inferred from their medication records alongside diagnosis codes and clinical care team notes. Hence, each participant presented with one index long-term condition and at least one other equally significant co-occurring condition. The long-term conditions spanned distinct multimorbidity clusters, including respiratory, cardio-metabolic, neurological, and musculoskeletal conditions [16].

### 2.2. Experimental Protocol

The experimental protocol has been detailed elsewhere [15]. Briefly, participants underwent a 2.5 h free-living monitoring session, performing their usual daily activities in their habitual environment (i.e., home, work, community, or outdoor), with no restrictions, and were also asked to complete specific tasks to ensure a range of walking behaviors, including outdoor walking, walking up and down slopes and stairs, and moving between rooms. Participants were equipped with a wrist-worn IMU, a lower-back IMU, and the multisensor INDIP system (INertial modules, DIstance Sensors and Pressure insoles).

### 2.3. IMU Data

Data from the target device were collected using a wrist-worn inertial sensor placed on the non-dominant wrist using an elastic strap. The sensor recorded triaxial acceleration at a sampling frequency of 100 Hz (range: ±8 g; resolution: 1 mg) and triaxial gyroscope data (range: ±2000°/s; resolution: 70 mdps), providing full 6-DoF motion data (221e S.r.l., Padova, Italy).

### 2.4. Reference Data

Reference data were collected using the INDIP system at 100 Hz, comprising two inertial measurement units (IMUs) affixed to the shoelaces (instep position), two distance sensors attached on the medial aspect of the lower shank with Velcro straps, and two pressure-sensing insoles. The INDIP central unit is a custom magneto-inertial board (221e S.r.l., Padova, Italy) containing a 3-axis accelerometer (±16 g), 3-axis gyroscope (±2000 dps), and 3-axis magnetometer (±50 G), with on-board storage, low-power processing, and wired/wireless transmission. Time-of-flight infrared distance sensors (range 0.2 m, 50 Hz) and force-sensitive resistor pressure insoles (16 sensors per insole, thickness 240 μm) were connected as sensing peripherals. The derivation of reference spatiotemporal gait characteristics using the INDIP system, in comparison to the stereophotogrammetry system, has been described and validated in detail previously [17]. Data from both wrist IMU and INDIP were synchronized using timestamps referred to a common clock (±1 sample).

### 2.5. Algorithm Selection and Optimization

We employed a combination of algorithms previously published in the literature, and novel improved or optimized versions (e.g., using adaptive/personalized thresholds) developed to enhance performance across diverse gait patterns in populations with equally significant long-term conditions. Improved or adaptive threshold versions were fine-tuned in the whole TVS population (*n* = 108) using 5-fold cross-validation. In each fold, 4/5 of the data were used to select the best-performing threshold based on the performance index (please see Section 2.10). The final threshold for each algorithm was chosen as the median of the five-fold-specific optimal thresholds to provide a robust, conservative estimate. A grid search identified the best-performing threshold/parameter combinations, to derive the improved or adaptive versions. Some algorithms were originally designed for a lower back sensor with specific axis orientations; we adapted them for wrist use by replacing axis-specific dependencies with the 3D accelerometer signal norm. Details of both original and adapted algorithms are presented in Table 1. The novel algorithm implementation and analysis scripts are publicly available on both GitHub and Zenodo (version 1.0.0; https://doi.org/10.5281/zenodo.16926413) [18]. Exceptions include the GSD Iluz, Ionescu, and ICD Shin and HK Lee algorithms, which have already been implemented in existing software and are publicly available [19].

(1) $$step\;length=A\;\times \;RMS\;\times \;\sqrt[{4}]{\left|maxmin\right|}\;+\;B$$

Novel step length estimation model based on Weinberg [34]; RMS: Root Mean Square of the acceleration signal between two consecutive ICs; maxmin: amplitude of the acceleration signal between two consecutive ICs; stride length = 2 × step length.

(2) $$step\;length=A\;\times \;RMS\;\times \;\sqrt[{3}]{\left|\mu \right|}\;+\;B$$

Novel step length estimation model based on Kim [35]; RMS: Root Mean Square of the acceleration signal between two consecutive ICs; μ: average of the acceleration signal between two consecutive ICs; stride length = 2 × step length.

(3) $$step\;length=A\;\times \;RMS\;\times \;\sqrt[{2.7}]{|\mu |\;\times \;\sqrt{1/\Delta t\;\times \;maxmin}}\;+\;B$$

Novel step length estimation model based on Bylemans [36]; RMS: Root Mean Square of the acceleration signal between two consecutive ICs; μ: average of the acceleration signal between two consecutive ICs; Δt: time difference between two consecutive ICs; maxmin: amplitude of the acceleration signal between two consecutive ICs; stride length = 2 × step length.

### 2.6. Performance Metrics

In Table S1 (online supplement), we present the predefined threshold ranges used to classify performance metrics as poor, acceptable, good, or excellent, based on values reported in previous validation studies [1,13]. The categories were defined such that “excellent” corresponds to performance similar to or better than the best-performing previously reported algorithms, “poor” falls below the worst-performing algorithms reported previously, and “acceptable” and “good” divide the range between “poor” and “excellent” evenly. For each algorithm and relative DMO, a customized validation and ranking approach was employed, following methods similar to those described previously [1]. Briefly, metrics representing benefits (e.g., accuracy) were used directly on a 0–1 scale, while cost metrics (e.g., absolute errors) were normalized to 0–1 using an exponential transformation; all metrics were then weighted and summed to produce an overall performance score.

### 2.7. GSD

The full recording was provided as an input for both systems. Each window of 0.1 s of each recording was classified as true positive (TP), false positive (FP), true negative (TN) or false negative (FN), while accuracy, recall (sensitivity), specificity, precision (positive predictive value) were calculated (see Online Supplement page 2). In addition, absolute errors and Intraclass Correlation Coefficients (ICC(2,1)) for the total accumulated duration of all gait sequences were calculated between the two systems. For the purposes of this study, gait sequences were defined as walking bouts containing at least two consecutive strides of both feet (e.g., R–L–R–L–R–L or L–R–L–R–L–R, where R/L denote right/left foot contacts). Consecutive bouts were separated by breaks of ≥3 s, whereas pauses ≤3 s were considered part of the same bout.

### 2.8. ICD

The refined gait sequences from the reference system were provided as input to the algorithms to allow comparisons with the reference. Each initial contact (IC) event in the reference walking bouts was classified as TP, FP, or FN by comparing the IC between the two systems within a tolerance window of ±0.25 s centered around the reference event. Recall (sensitivity), precision (positive predictive value), absolute and relative error (normalized to average step duration per walking bout) were used as performance metrics.

### 2.9. SL

For stride length estimation both the reference WBs and ICs were provided to allow for reliable comparisons. The measures used included relative errors, absolute errors and ICC(2,1).

### 2.10. Index-Based Algorithm Ranking

An adapted version of the ranking methodology (described previously [1,37]) was employed to compare algorithm performance via a weighted decision matrix. Performance measures were classified as either benefits (e.g., accuracy, recall, specificity, precision, ICC) or costs (e.g., absolute and relative errors), with cost normalized using an exponential transformation to ensure compatibility. Each metric was weighted according to its relevance to validity assessment using customized weights presented in Tables S2–S4 (online supplement). A performance index (ranging from 0 (worst) to 1 (best)) was calculated as a weighted mean, enabling algorithm ranking.

## 3. Results

A total of 28 participants experienced a median of three long-term conditions, with a range of two to four co-occurring long-term conditions of equal importance. Demographic and clinical characteristics are presented in Table 2.

### 3.1. Performance Metrics of Algorithms

#### 3.1.1. Gait Sequence Detection

We report in Table 3 the GSD algorithm performance metrics. Performance metrics for the nine gait sequence detection algorithms ranged from average to excellent. In the nine evaluated algorithms, recall (sensitivity) ranged from 0.31 in MacLean to 0.64 in Ionescu adaptive, indicating substantial variation in the ability to correctly detect walking bouts. Specificity was consistently high, ranging from 0.90 (Iluz) to 0.98 (Keren improved and adaptive), reflecting strong performance in correctly identifying non-walking periods. Accuracy varied between 0.81 (MacLean) and 0.92 (Kheirkhahan, Ionescu), while precision (positive predictive value) ranged from 0.38 (MacLean) to 0.63 (Kheirkhahan, Ionescu). Ionescu adaptive, Hickey, and Iluz methods overestimated the total gait sequence duration, whereas the remaining algorithms underestimated it. The ICC(2,1) ranged from 0.37 (Iluz) to 0.64 (Kheirkhahan).

Among the evaluated methods, Kheirkhahan achieved the highest overall performance, exhibiting high accuracy (0.92), specificity (0.96), and precision (0.63), alongside the highest ICC (0.64). The second-best performance was observed in the Ionescu method, which showed comparable accuracy (0.92), precision (0.63), and recall (0.55), but slightly lower ICC (0.60).

A comparison of the performance index between original and novel, or refined algorithms is shown in Figure S5 in the online supplement.

#### 3.1.2. Initial Contact Detection

Table 4 summarizes the performance metrics of the evaluated ICD algorithms. Sensitivity (recall) ranged from good to excellent (0.69 (Gu adaptive) to 0.82 (McCamley)), while positive predictive value (precision) varied between 0.65 (Micó-Amigo wrist) and 0.82 (Shin). Absolute timing errors ranged from 0.09 s (12% relative error) for ShinIC to 0.13 s (19% relative error) for HKLeeIC. Among the algorithms, Shin wrist achieved the highest overall performance index of 0.85, reflecting its balanced precision (0.82) and recall (0.77). The second-best performance was observed for McCamley wrist with a performance index of 0.84, demonstrating the highest recall (0.82) but slightly lower precision (0.77). The third-best performance was achieved by the Zijlstra method, achieving a performance index of 0.83, with balanced recall (0.78) and precision (0.77).

**Figure 1:**
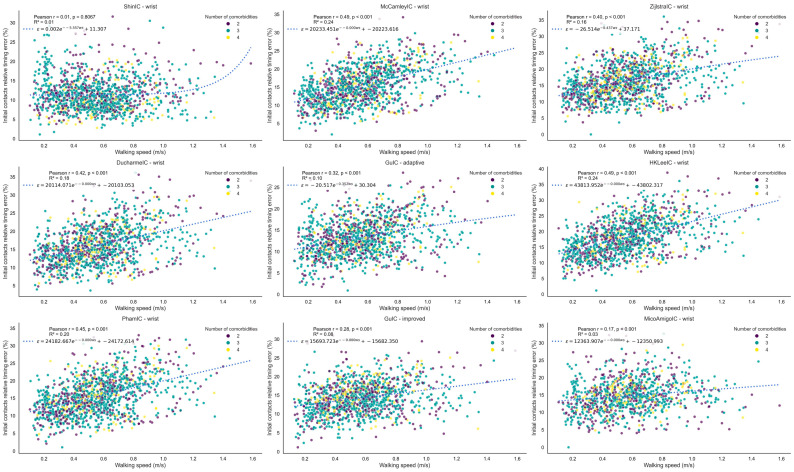
Plot of relative initial contact timing error versus walking speed for all initial contact detection algorithms. Each point reflects the relative error and corresponding walking speed calculated from a single walking bout. The figure illustrates how the measurement error of the wearable system’s initial contact detection varies with walking speed across all algorithms evaluated. The fitted curve represents an exponential decay, *ϵ* = *a* *e*^− *b* x^ + *c*, derived via non-linear least squares (parameters *a*, *b*, and *c*). The *R*^2^ value indicates the goodness-of-fit between the data points and the fitted curve.

**Figure 2:**
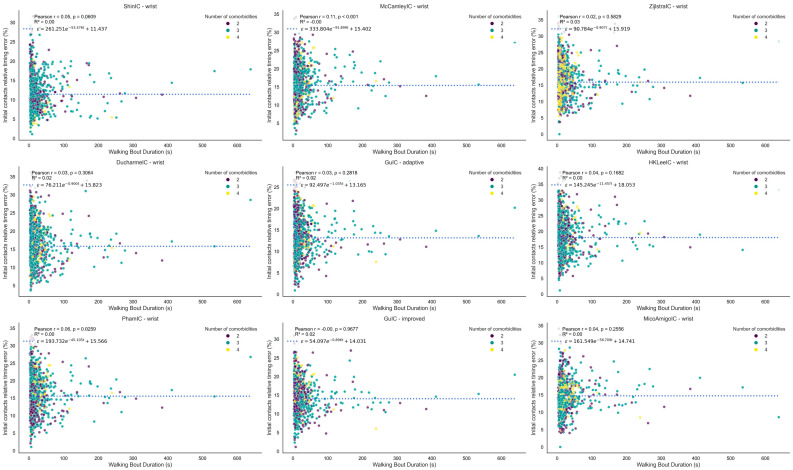
Plot of relative initial contact timing error versus walking bout duration for all initial contact detection algorithms. Each point reflects the relative error and corresponding walking bout duration calculated from a single walking bout. The figure illustrates how the wearable system’s measurement error in initial contact detection varies with walking bout duration across all algorithms evaluated. The fitted curve represents an exponential decay, *ϵ* = *a* *e*^− *b* x^ + *c*, derived via non-linear least squares (parameters *a*, *b*, and *c*). The *R*^2^ value indicates the goodness-of-fit between the data points and the fitted curve. Each point is color-coded based on the number of co-occurring long-term conditions.

The relative initial contact timing errors are presented versus walking speed (Figure 1) and walking bout duration (Figure 2).

#### 3.1.3. Stride Length

Performance metrics for stride length algorithms are presented in Table 5. The Weinberg and Bylemans algorithm versions consistently overestimated stride length, while the Kim algorithm versions consistently underestimated it. The Weinberg wrist and wrist adaptive versions showed the best performance, with absolute errors of 0.19 m [0.16, 0.21] m and relative errors of 31 [25, 37] for both versions. The third best-performing method was the Bylemans adaptive version incorporating foot length, with an absolute error of 0.20 m [0.18, 0.22] m and a relative error of 32 [27, 38].

**Figure 3:**
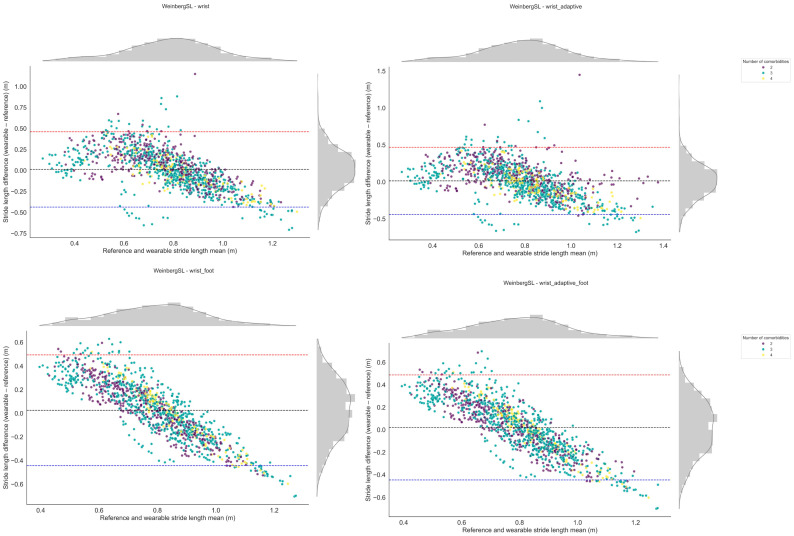
Bland–Altman plots illustrating the agreement and potential bias between sensor-derived and reference stride length measurements for the Weinberg algorithm. Each point is derived from a single walking bout, plotted as the difference between methods against their mean. Distributions of the stride length differences and means are shown along the top and right margins, respectively. Differences were calculated as wearable—reference, such that positive values indicate overestimation by the wearable and negative values indicate underestimation. Each point is color-coded based on the number of co-occurring long-term conditions.

**Figure 4:**
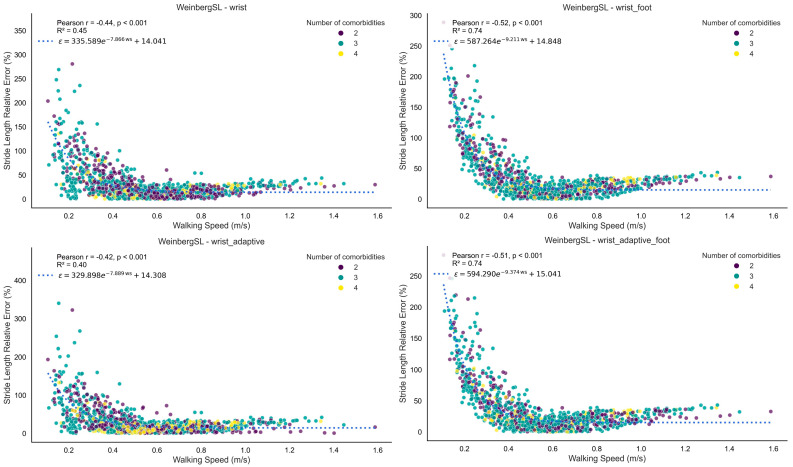
Plot of relative stride length error (%) versus walking speed for the Weinberg algorithm. Each point reflects the relative error and corresponding walking speed calculated from a single walking bout. The figure illustrates how the wearable system’s measurement error varies with walking speed. The fitted curve represents an exponential decay, *ϵ* = *a* *e*^− *b* x^ + *c*, derived via non-linear least squares (parameters *a*, *b*, and *c*). The *R*^2^ value indicates the goodness-of-fit between the data points and the fitted curve. Each point is color-coded based on the number of co-occurring long-term conditions.

**Figure 5:**
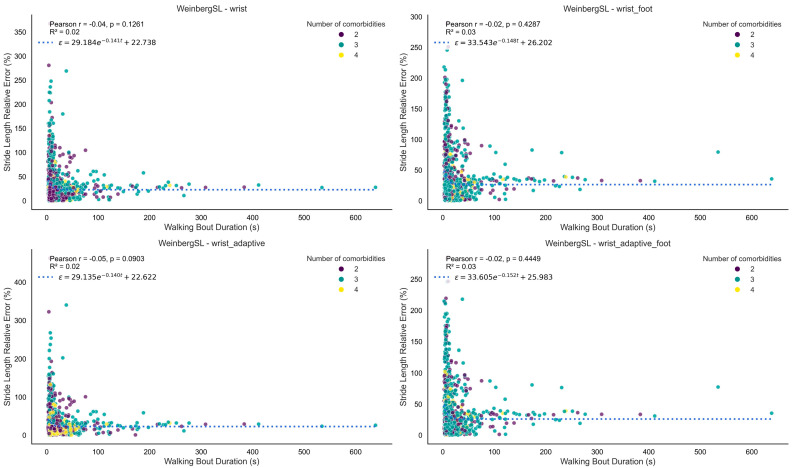
Plot of relative stride length error (%) versus walking bout duration for the Weinberg algorithm. Each point reflects the relative error and corresponding walking bout duration calculated from a single walking bout. The figure illustrates how the wearable system’s measurement error varies with walking bout duration. The fitted curve represents an exponential decay, *ϵ* = *a* *e*^− *b* x^ + *c*, derived via non-linear least squares (parameters *a*, *b*, and *c*). The *R*^2^ value indicates the goodness-of-fit between the data points and the fitted curve. Each point is color-coded based on the number of co-occurring long-term conditions.

Figure 3 shows Bland–Altman plots comparing stride length from all four versions of the Weinberg algorithm to the reference. Figure 4 presents the relative stride length error versus walking speed for all versions of the Weinberg algorithm. Figure 5 shows the relative stride length error in relation to walking bout duration for the same algorithm. Supplementary Figure S1 shows similar Bland–Altman plots for the Bylemans algorithm, while Figures S3 and S4 present its relative stride length error versus walking speed and walking bout duration, respectively.

## 4. Discussion

### 4.1. Main Findings

This study presents the first comprehensive technical validation of gait detection algorithms for a single wrist-worn device in people with multiple long-term conditions (MLTCs), conducted in a real-world setting to ensure ecologically valid assessment.

We successfully extracted spatial and temporal mobility outcomes, including gait sequence detection (GSD), initial contact detection (IC), and stride length estimation (SL), from a wrist-worn sensor in a cohort with MLTCs. A major contribution of this study is the successful estimation of stride length from a single wrist-worn sensor with high performance, representing a significant advance in spatial gait analysis from a highly accessible sensor location. Several novel algorithmic adaptations were introduced and tested, specifically tailored to the variability and signal complexity encountered in diverse gait patterns from a cohort with diverse disease entities. To the best of our knowledge, this is the first detailed real-world validation of wrist-derived GSD, ICD, and SL in a multimorbid sample, with 4200 min of continuous monitoring from 28 participants.

### 4.2. GSD

The three top-performing GSD methods were the Kheirkhahan, Ionescu, and Keren, the latter being the only method originally developed specifically for a wrist-worn device. Following fine-tuning, performance improved by 22.6% in the Kheirkhahan, by 25% in the Ionescu, and by a modest 3% in the Keren. Among the remaining methods, Hickey performed better than Iluz and MacLean wrist versions. While these methods were outperformed by the top three, they nonetheless achieved good recall and precision in detecting gait sequences.

When compared to results in single-disease cohorts reported by Kluge et al. [13], performance metrics (e.g., specificity, recall) in Kheirkhahan, Ionescu, and Hickey algorithms was similar or slightly higher, whereas Iluz performed worse in the present study. We attribute the improved metrics in the former algorithms to the extended fine-tuning implemented here, and the reduced performance of Iluz to the increased heterogeneity of our sample, which included diverse multimorbid populations rather than focusing on a single disease condition. However, the best-performing wrist-based model in our study (Kheirkhahan) underperformed the lower-back-derived performance metrics (sensitivity and precision) reported in the aforementioned sample for participants with a single long-term condition [1], likely reflecting that the earlier validation was disease-specific and the sensor was placed on the lower-back, which is a position with fixed axes, whereas the present work targeted a multimorbid sample. In contrast, accuracy and specificity were similar between studies.

Adaptive or personalized variants—where thresholds or constants were dynamically adjusted to individual signal characteristics—were implemented in Ionescu and Keren methods. In both methods the adaptive versions yielded improved performance compared to the original methods but similar to the fine-tuned adaptive variants. However, we anticipate that in new samples of multimobidity, the personalized nature of adaptive methods may enable superior generalization and improved performance compared to their fixed-threshold counterpart.

### 4.3. ICD

The highest-performing ICD method was the Shin; it achieved a performance index of 0.85 and demonstrated stable error levels across walking speeds. The McCamley method ranked second (performance index: 0.84). The Zijlstra method ranked third, yielding a performance index of 0.83 and an improvement of almost 23% compared to the original method. The Ducharme method achieved a performance index of 0.82. The Gu, HKLee, Pham, and Micó-Amigo methods demonstrated slightly lower performance, with Micó-Amigo being the only approach with an index below 0.80 (0.78). Across all methods, the absolute timing error ranged from 0.09 s for the best-performing method to 0.13 s. When compared with prior reports in single-disease cohorts using a single lower-back sensor, our fine-tuned wrist adaptations achieved comparable recall, precision, as well as timing errors, across all algorithms [1]. This strong performance, despite the use of wrist-worn sensor, is likely due to the systematic fine-tuning process and the development of novel algorithmic variants. The introduction of novel algorithmic versions and fine-tuned thresholds was efficacious, while adaptive version in one algorithm (Gu) with thresholds dynamically adjusted to individual signal patterns slightly outperformed the respective fixed-threshold version. However, we expect that adaptive versions will perform better in new samples given that they are adaptive to the individual signal and gait pattern.

Most algorithms exhibited higher errors at faster walking speeds (Figure 1), but Shin and Micó-Amigo methods maintained stable error levels across all speeds, making them more robust for diverse gait patterns. The robustness of the Shin and Micó-Amigo algorithms across walking speeds likely reflects their reliance on phase-based (Shin) or time-aligned template-matching (Micó-Amigo) detection strategies, which are less sensitive to amplitude variation and step frequency changes compared to the predominantly threshold-based approaches used by other methods. This poorer performance at higher speeds may reflect altered arm-swing or other upper-limb movements and increased high-frequency components. Regarding walking bout duration (Figure 2), all methods demonstrated robustness, with no notable degradation in performance across short or long bouts. For the exponential fits, Pearson r values were close to zero and R^2^ values were very low (<0.04), indicating no meaningful correlation or trend. Similarly, the number of co-occurring long-term conditions (within the limited range of 2–4) did not exhibit any discernible influence on error across different walking speeds or bout durations. The absence of patterns may be attributed to the limited range of co-occurring conditions in the sample. The only noticeable pattern was a confined increase at very small walking bout durations (<4 s), after which performance stabilized.

### 4.4. SL Estimation

The top-performing stride length estimation method consisted of the Weinberg algorithm, the fine-tuned wrist version achieved the highest performance index (0.72), all versions exhibited minimal bias, and most values fell within the ±1.96 SD limits in Bland–Altman analysis. Adaptive versions incorporated the root mean square (RMS) of acceleration between consecutive initial contacts, based on the theoretical assumption that RMS magnitude increases with step length. In the foot length-augmented version, an additional constant term proportional to the participant’s foot length was added (parameter B, Table 1). The latter parameter was not fine-tuned in the wrist and adaptive versions to avoid overfitting to the study population, while the inclusion of the foot length added to the intensity-based model is biomechanically justified given the proportional relationship between foot size and stride length. All four versions of the Weinberg method exhibited a biphasic bias–error relationship in the Bland–Altman analysis; each model overestimated stride length at shorter strides, underestimated at longer strides, and agreed only at intermediate stride lengths, which comprised the majority of observations given the approximately normal distribution of values in the plots. This inconsistency suggests that over- and underestimation may balance out across the measurement range. Notably, in the foot-length-augmented versions, the trend was more pronounced, likely because adding foot length as a parameter to the intensity-based model amplifies magnitude-dependent scaling effects, increasing proportional bias and thus exaggerating over- or underestimation at the extremes.

The second-highest-performing method was Bylemans. Its adaptive version also incorporated RMS between steps, improving robustness to speed variation. Similar to Weinberg, foot length-augmented versions were tested and the best performance metrics were achieved by the adaptive version incorporating foot length (0.66), while the worst metrics were observed in the wrist version (0.55). The Bylemans versions did not exhibit a consistent trend in the Bland–Altman plots, indicating that their estimation errors were randomly distributed across the measurement range without systematic over- or underestimation. However, the wrist foot-length-augmented model showed a biphasic pattern similar to the Weinberg versions. Among the three trend-free versions, the wrist adaptive model displayed a higher number of outliers and the worst metrics overall, suggesting greater variability in estimation accuracy and reduced reliability across stride lengths compared to the others. Across both methods (i.e., Bylemans, Weinberg), relative error was higher at lower walking speeds but decreased and stabilized rapidly across the observed range (Figure 4 and Figure S3). Bylemans’ adaptive version appeared less speed-sensitive than the other variants. Neither Weinberg nor Bylemans showed any association between relative error and walking bout duration. The range of co-occurring long-term conditions did not appear to affect error patterns, possibly due to the small range of co-occurring conditions included in our sample.

The Kim algorithm ranked third. Its mathematical formulation proved less effective in adjusting stride length values within walking bouts, reflected in the Bland–Altman plot (Figure S2), where stride length estimates repeatedly cluster along discrete values, indicating the algorithm’s inability to capture natural variability. Hence, we suggest the use of Weinberg and Bylemans methods.

When comparing our relative stride length errors to those reported from a lower-back sensor in single-disease populations using biomechanical models [1], results were broadly comparable, though some cohorts showed lower errors (25–30% vs. 31% in our study). Machine learning (ML) models on lower-back sensor reported smaller errors (Root Mean Squared Error (RMSE) 6–7 cm) [38], while our best intensity-based methods reached absolute errors of 19 cm. For wrist-worn sensor, recent ML models achieved RMSE values of 14–18.3 cm and Mean Absolute Error (MAE) of 11.6–14.5 cm [39], slightly outperforming our intensity-based models. Direct comparisons remain limited by differing error metrics, but these findings point to a performance gap between biomechanical, intensity-based, and machine learning approaches. Given that wrist-worn IMU axes are not fixed in anatomical space, traditional biomechanics-based models cannot be applied directly; instead, the fine-tuned intensity-based approaches presented here—incorporating adaptive RMS and foot length augmentation—offer a computationally efficient alternative requiring minimal processing power and suitable for real-time implementation. While thresholds optimized in this study may not generalize across all populations, these methods represent a significant advance in spatial gait analysis from wrist-worn sensor. Future work will aim to build on this foundation using AI-based models that can further account for the complexities of human movement, including non-linear gait adaptations and context-dependent variability.

### 4.5. Algorithm Performance and Gait Pattern Diversity

While some algorithms achieved consistently high validity across the full sample, others showed performance limitations. Higher-performing algorithms were able to detect and accommodate the diversity of gait patterns present in this multimorbid population including different disease entities such as cardiovascular disease, chronic obstructive pulmonary disease, Parkinson’s disease, hypertension, arthritis, and proximal femur fracture, where individuals often exhibit multiple, overlapping gait impairments [1,40,41]. This high degree of variability—both across different individuals and within the same individual—is a defining feature of real-world multimorbidity. Algorithms that are unable to accommodate this complexity are therefore not suitable for use in such populations.

### 4.6. Implications for Technically Valid DMOs in Multimorbidity

DMOs provide objective, real-world measures of functional status and performance. In populations with multimorbidity, DMOs can serve as a unifying biomarker, reflecting the combined impact of multiple conditions on physical function. They can support early diagnosis by detecting subtle functional decline associated with frailty, sarcopenia, or specific long-term conditions, and aid in risk stratification for falls, hospitalization, and mortality [42]. DMOs can also monitor disease progression or treatment response, capturing improvements or deterioration following interventions, and guide personalized interventions [43], particularly in individuals living with multiple long-term conditions where functional decline is common.

### 4.7. Strengths and Limitations

A major contribution of this study is the successful estimation of stride length from wrist-worn sensor with high performance, representing a significant advance in spatial gait analysis from a highly accessible sensor location. Our work expands prior validation efforts, which have predominantly focused on lower-back sensor and single-disease cohorts, by demonstrating that accurate and reliable mobility outcomes can be extracted from wrist-worn devices in populations with diverse, multiple long-term conditions.

Importantly, we have introduced stride length estimation signal processing algorithms specifically tailored and validated for wrist-worn sensors, based on dead reckoning principles commonly used in wearable navigation systems, thereby complementing existing spatial–temporal mobility outcomes. Additionally, a recent pre-print presents a novel deep learning approach for stride length estimation from wrist-worn monitors [39], based on an extension of an existing deep learning model for gait sequence detection [44], representing an accurate and distinct methodology compared to purely signal-processing approaches. Unlike traditional biomechanics-based models, which are computationally complex [30] and less suited for real-time use, the presented stride length algorithms are computationally efficient, making them well-suited for online applications. However, because these algorithms were fine-tuned to a specific multimorbid sample, their high performance may not generalize to other populations with different gait patterns, a limitation akin to overfitting in machine learning models, contrasting with more generalizable biomechanics-based approaches. Hence, we emphasize the use of the novel adaptive and foot length-augmented versions, which incorporate individual-specific adjustments to improve robustness and applicability across diverse populations. All algorithms evaluated here are openly available and will be integrated into an efficient processing pipeline designed to support real-world mobility monitoring and longitudinal data aggregation in clinical and research settings. This approach facilitates scalable deployment of wearable technology for remote health assessment and monitoring in complex multimorbid populations.

Nevertheless, several limitations should be acknowledged. The analysis was based on a pre-existing dataset designed for single long-term conditions, and the small multimorbid subsample precluded stratification by specific disease combinations or functional impairment severity. Study participants had a median of three co-existing long-term conditions, spanning over 16 different diagnoses across at least seven body systems (cardiovascular, pulmonary, musculoskeletal, digestive, mental health, neurological, and endocrine). This specific cohort composition is thus expected to shape our findings, as multimorbidity typically leads to greater reductions in functional capacity than a single condition alone. This arises from the cumulative burden, where each condition contributes symptoms such as breathlessness, fatigue, and pain that compound disability, and from systemic interactions, whereby diseases affecting different systems can exacerbate one another’s effects (e.g., COPD and heart failure amplifying physical functioning intolerance) [42]. Our cohort reflects the complexity of multimorbidity seen in many patients, supporting the broader relevance of our findings. However, generalizability may be limited by the specific mix and severity of conditions represented [8]. Given the wide spectrum of gait alterations associated with different pathologies and disease severity, future investigations (ISRCTN25008143) should include carefully phenotyped multimorbid clusters and matched reference standards to further validate and refine these algorithms, enabling targeted clinical applications and personalized monitoring strategies. Furthermore, the reference data were derived from refined gait sequences, whereby micro walking bouts separated by <3 s were merged. This approach may disadvantage algorithms capable of detecting gait at the micro-bout level, leading to lower performance metrics in our evaluation. Furthermore, we note that, because the validation sample of 28 participants was part of the full 108-participant sample on which the threshold optimization was performed, the selected thresholds may partially reflect characteristics of the reported subsample; nevertheless, the 5-fold cross-validation and median-of-fold selection provides a robust and conservative estimate of thresholds in the fine-tuning process, mitigating overfitting to individual subjects.

This study focused on signal processing methods to maximize granularity and interpretability at the sample frequency level; hence, it did not include ML approaches. Current published AI/ML gait models lack the necessary temporal resolution for direct comparison with our reference methods, which support precision (gait detection) per sample, as opposed to published models including gait detection at 10 s windows [44] or total ambulation time per recording periods [45]. In addition, we focused on signal-processing algorithms because they can be open access, mechanistic, and fully transparent, allowing for applications such as digital biomarkers or endpoints and the provision of fully inspectable evidence of algorithmic validity and properties. While AI models may achieve higher accuracy, particularly when integrated with biomechanically sound pipelines, they often lack the same level of interpretability and transparency, which can limit their immediate suitability for regulatory use in clinical trials. A recent self-supervised learning model demonstrates gait detection including stride length and walking speed with granularity at the level of 10 s or longer walking bouts, highlighting the potential of AI approaches for coarser temporal resolutions [39]. Future work will incorporate ML-based models for high-granularity gait detection from wrist sensors, leveraging the strong performance of the validated algorithms presented here while aiming to capture additional variability in multimorbid populations with diverse gait characteristics.

## 5. Conclusions

This study successfully developed, fine-tuned, and validated algorithmic approaches for extracting key mobility outcomes (gait sequence detection, initial contact timing, and stride length estimation) from wrist-worn sensor in a diverse, multimorbid population in real-world conditions. Our findings demonstrate that these methods perform robustly across diverse gait patterns, surpassing or matching the accuracy of existing algorithms originally designed for other sensor locations or single-disease cohorts. In addition, novel intensity-based, computationally efficient methods for stride length estimation exemplify the scalability of this approach using widely accessible wearable technology. This paves the way for improved remote health assessment and personalized rehabilitation strategies in diverse clinical populations.

## Figures and Tables

**Table 1:** Gait sequence detection (GSD), initial contact event detection (ICD), and stride length estimation (SL) algorithm descriptions, adaptations, modified and novel versions.

Algorithm	Description	Adaptations for Wrist-Worn Sensor and Improved/Novel Versions	Thresholds per Version (in g-Unit for Simplicity):
**GSD**			
Hickey [20]	Identifies bouts of walking using window-based calculations of the acceleration signal variability and orientation thresholds. Includes resampling, gravity removal, axis correction, and Butterworth filtering.	Gravity is removed from all axes before computing the acceleration norm. The “thresholdstill” is fine-tuned for the wrist-worn position. The original upright-position threshold, based on vertical acceleration from a lower-back sensor, is replaced by a maximum activity threshold applied to the norm. This threshold corresponds to the 100th percentile of wrist acceleration during walking bouts in the TVS dataset (*n* = 108), excluding high-intensity activities that may meet other walking variability criteria.	wrist * thresholdstill = 0.1 thresholdupright = 9.5
Kheirkhahan [21]	Identifies walking bouts using activity counts from triaxial acceleration. Data are preprocessed, segmented into overlapping windows, and windows meeting criteria are marked as walking.	The optimized wrist version uses the acceleration norm and improved threshold, and window size fine-tuned for the wrist worn position.	wrist * threshold = 0.58 win_size = 9
MacLean [22]	Identifies walking bouts using a threshold-based algorithm. The signal is filtered, centered, and the norm is used. A binary activity signal is generated and smoothed to identify continuous active periods. Short inactive gaps between active segments are merged, and candidate bouts are evaluated against signal intensity and duration criteria.	Since the acceleration norm is used already, the thresholds have been fine-tuned for the wrist-worn position.	wrist * threshold_binary = 0.11 gap_threshold = 0.4 gap_index = 0.1 walk_threshold = 0.5 walk_index = 0.05
Keren [23]	Identifies walking bouts using a multi-step algorithm applied. The norm of the signal is filtered and detrended. Gait-like windows are detected based on peak presence, signal variability, dominant frequency, and autocorrelation regularity. Conditions are evaluated in overlapping windows, and consecutive valid segments are merged into walking bouts.	The improved version includes fine-tuned thresholds. An adaptive version has been introduced using a dynamic threshold based on a percentile of the acceleration signal, rather than a fixed value.	improved * threshold = 0.08 threshold_sd = 0.07 adaptive * threshold_percentile = 84 threshold_sd = 0.07
Ionescu [24]	Identifies walking bouts by detecting steps from the low-pass filtered acceleration norm. Peaks above a threshold indicate steps, which are grouped into gait sequences using an adaptive step duration threshold. In addition, an adaptive version sets the step threshold based on a percentile of peak amplitudes in detected active periods.	Since the acceleration norm is used already, the thresholds of both the fixed and adaptive versions have been fine-tuned for the wrist-worn position.	wrist * active_signal_threshold = 0.31 wrist_adaptive* active_signal_fallback_threshold = 0.4 percentile = 31
Iluz [25]	Identifies walking bouts using a frequency-based approach applied to vertical and anterior–posterior acceleration signals. First, these signals are band-pass filtered. Then, a convolution with a sine wave is performed, and local maxima from this convolution are detected to define gait regions.	To adapt the algorithm for wrist-worn data, gravity removal is applied per axis at the start, activity is detected using the acceleration norm, standing and orientation change checks are removed, and peak detection is performed only once.	wrist * std_threshold = 0.06 step_threshold = 0.84
**ICD**			
Ducharme [26]	Detects initial contact events using the tri-axial accelerometer norm. The signal is first detrended by mean subtraction, then resampled to 80 Hz to apply a fourth-order Butterworth bandpass filter (0.25–2.5 Hz). Peaks above a specified threshold are identified as initial contacts using a standard peak detection function. The detected peak indices are then rescaled to the original sampling frequency.	The algorithm was designed for a lower-back sensor but already operates on the norm of tri-axial acceleration. To adapt it for wrist-worn sensor, the detection threshold has been fine-tuned.	wrist * threshold = 0.01
Gu [27]	Detects initial contacts using peak detection and multi-stage filtering. It segments the signal, identifies local maxima, and applies thresholds on peak magnitude, periodicity, similarity, and continuity to improve robustness.	The algorithm was designed for use with a wrist-worn sensor. The thresholds have been fine-tuned. In addition, a novel adaptive version has been introduced based on percentiles of the acceleration signal for the magnitude threshold.	improved * k = 2 period_min = 25 period_max = 120 sim_thres = −0.7 var_thres = 0.0005 mag_thres = 1.1 adaptive * k = 2 period_min = 25 period_max = 110 sim_thres = −0.7 var_thres = 0.005 × (9.81)^2^ mag_thres = 70
Shin [28]	Detects initial contacts on the norm of the acceleration signals. A sliding window sum reduces noise in the acceleration norm. A differencing step acts as a high-pass filter to remove gravity. Initial contacts are then identified as zero-crossings on the positive slope.	The algorithm was designed for a lower-back sensor but already operates on the norm of tri-axial acceleration; hence, it is used as-is.	original *
Lee [29]	Detects initial contacts by preprocessing the acceleration norm using low-pass filtering, detrending, Savitzky–Golay smoothing, and Gaussian smoothing, followed by a continuous wavelet transform to enhance step features. Morphological filters are then applied, and initial contact events are detected as maxima between zero-crossings.	The algorithm was designed for a lower-back sensor but already operates on the norm of tri-axial acceleration; hence, it is used as-is.	original *
Zijlstra [30]	Detects initial contacts by preprocessing the acceleration signal with detrending and low-pass filtering to isolate gait-related components. Initial contacts are then identified either by detecting positive-to-negative zero crossings or by locating peak maxima between zero crossings.	The algorithm was designed for a lower-back sensor and the anteroposterior axis was used; hence, in the wrist version the acceleration norm was used. The peak detection method as well as the cutoff for the Butterworth filter were fin- tuned for use in wrist-worn IMUs.	wrist * cutoff = 2.5 peak detection method = “peak”
Micó-Amigo [31]	Detects initial contacts by estimating step periodicity via autocovariance and spectral analysis to define a subject-specific template. A template-matching approach based on dynamic time warping (DTW) identifies high-similarity segments through normalized correlation and variance. Peaks in the resulting similarity signal are selected as initial contacts.	Adapted for wrist-worn sensor by removing the gravity component from the 3-axis acceleration signal using a Butterworth filter, followed by computing the signal norm replacing the original lower-back anteroposterior axis. Two new parameters, peakdistance and peakdistance_coef, have been introduced and fine-tuned for wrist-worn sensor. These control the minimum spacing between peaks in the acceleration and similarity signals, respectively, and were optimized to improve detection accuracy.	wrist * peakdistance = 1.1 peakdistance_coef = 1.0 shiftfactor = 0.15 factorlimit = 2 event_offset = 5
McCamley [32]	Detects initial contacts by downsampling (50 Hz) and preprocessing the acceleration signal with detrending and a low-pass Butterworth filter (20 Hz), followed by cumulative trapezoidal integration. The integrated signal is smoothed using a continuous wavelet transform (CWT) and upsampled back to the original sampling rate. Initial contacts are identified as local minima. Detected events are then filtered to remove those occurring less than 0.25 s apart or isolated beyond 2.25 s from neighboring events.	The algorithm was designed for a lower-back sensor using the vertical (inferosuperior) acceleration axis. For the wrist version, the acceleration norm is used instead. In addition, the wavelet center frequency is dynamically set using the signal’s dominant frequency to enchase sensitivity to individual gait patterns.	wrist * cwt_method = “adaptive”
Pham [33]	Detects initial contacts by upsampling (128 Hz) and preprocessing the acceleration signal with detrending, low-pass Butterworth filtering (10 Hz), and cumulative trapezoidal integration. The smoothed signal is further processed using a continuous wavelet transform (CWT). The resulting signal is detrended again, and local minima are detected. Peaks are retained only if their magnitude exceeds a specified percentage of the average peak amplitude.	The algorithm was originally designed for a lower-back sensor using the anteroposterior axis. For the wrist version, the acceleration vector norm was used instead. The peak detection threshold percentage was fine-tuned to optimize performance for wrist data. In addition, the wavelet center frequency is dynamically set using the signal’s dominant frequency to enchase sensitivity to individual gait patterns.	wrist * percentage_thresh = 0.02 cwt_method = “adaptive”
**SL**			
Weinberg [34]	Step length is estimated using an intensity-based method. The acceleration signal is preprocessed by computing the Euclidean norm and applying a low-pass Butterworth filter (2 Hz). Step length is calculated between consecutive initial contacts using the formula $step\;length\;=\;A\;\times \sqrt[{4}]{\left|maxmin\right|}\;+\;B$. Values are interpolated to per-second resolution; stride length is twice the step length.	Since the original algorithm was developed within the framework of inertial dead reckoning systems, finely tuned versions are provided in this paper. Additionally, adaptive versions are introduced, which utilize the root mean square (RMS) of acceleration between consecutive initial contacts multiplied by a finely tuned constant threshold (see Equation (1)). Furthermore, foot length-augmented variants are introduced, where an additional term based on individual foot length (in cm) is incorporated into the model to personalize stride length estimation.	wrist * A = 0.62 B = 0 wrist_footlength * A = 0.21 B = foot length (cm) wrist_adaptive * A = 0.60 B = 0 wrist_adaptive_footlength * A = 0.20 B = foot length (cm)
Kim [35]	Step length is estimated using an intensity-based method. Step length is calculated between consecutive initial contacts using the Euclidean norm and the formula $step\;length\;=\;A\;\times \;\sqrt[{3}]{\left|\mu \right|}\;+\;B$. Values are interpolated to per-second resolution; stride length is twice the step length.	Since the original algorithm was developed within the framework of inertial dead reckoning systems, finely tuned versions are provided in this paper. Additionally, adaptive versions are introduced, which utilize the RMS of acceleration between consecutive initial contacts multiplied by a finely tuned constant threshold (see Equation (2)). Furthermore, foot length-augmented variants are introduced, where an additional term based on individual foot length (in cm) is incorporated into the model to personalize stride length estimation.	wrist * A = 0.35 B = 0 wrist_footlength * A = 0.10 B = foot length (cm) wrist_adaptive* A = 0.35 B = 0 wrist_adaptive_footlength * A = 0.10 B = foot length (cm)
Bylemans [36]	Step length is estimated using an intensity-based method with signal preprocessing. Acceleration data are high-pass filtered and smoothed using a moving average. Step length is calculated between consecutive initial contacts using the formula $step\;length\;=\;A\;\times \;\sqrt[{2.7}]{|\mu |\;\times \;\sqrt{1/\Delta t\;\times \;maxmin}}\;+\;B$; stride length is twice the step length.	Since the original algorithm was developed within the framework of inertial dead reckoning systems, finely tuned versions are provided in this paper. The preprocessing of the signal has been improved in the current implementation by replacing the original custom IIR high-pass filter with a 4 Hz 4th-order Butterworth filter for improved signal fidelity and reproducibility. Additionally, adaptive versions are introduced, which utilize the RMS of acceleration between consecutive initial contacts multiplied by a finely tuned constant threshold (see Equation (3)). Furthermore, foot length-augmented variants are introduced, where an additional term based on individual foot length (in cm) is incorporated into the model to personalize stride length estimation.	wrist * A = 2.30 B = 0 wrist_footlength * A = 0.75 B = foot length (cm) wrist_adaptive * A = 9.15 B = 0 wrist_adaptive_footlength * A = 3.46 B = foot length (cm)

* naming of version present in the open access algorithms in Python; GSD: gait sequence detection; ICD: initial contact detection; SL: stride length estimation.

**Table 2:** Sociodemographic and clinical characteristics of 28 people with co-occurring long-term conditions.

Variable	*n* = 28
Age, mean (SD)	70.4 (10.7)
Sex, Female *n* (%)	12 (43%)
Height (cm), mean (SD)	168.9 (9.2)
Weight (Kg), mean (SD)	77.8 (16.8)
BMI (Kg/m^2^), mean (SD)	27.4 (6.3)
MoCa score, median (Q1–Q3)	26 (21–28)
VAS score, GeneralPain, median (Q1–Q3)	6 (3–26)
VAS score, Walking Pain, median (Q1–Q3)	8 (2–38)
LLFDI score, median (Q1–Q3)	58 (49–67)
Fall History, Yes *n* (%)	11 (39%)
Walking aid use, *n* (%)	
One cane/crutch	4 (14%)
Rollator	3 (11%)
Walker	1 (4%)
Other	1 (4%)
Number of co-occurring long-term conditions, median (range)	3 (2–4)
Cardiovascular Disease	14 (50%)
Chronic Obstructive Pulmonary Disease	8 (29%)
Lung Disease (other than COPD)	4 (14%)
Hypertension	20 (71%)
Arthritis	3 (11%)
Gouty arthritis	2 (7%)
Depression	3 (11%)
Hyperlipidemia	5 (18%)
Multiple Sclerosis	4 (14%)
Type 2 Diabetes	3 (11%)
Parkinson’s Disease	8 (29%)
Proximal Femur Fracture	6 (21%)

SD: standard deviation; Q1–Q3: first and third Quartiles; BMI: body mass index; MoCa: Montreal Cognitive Assessment; VAS: Visual Analogue Scale; LLFDI: Late-Life Function and Disability Instrument; COPD: Chronic Obstructive Pulmonary Disease.

**Table 3:** Gait sequence detection algorithm performance metrics ranked by performance index, with all metrics and error measures reported as mean values with 95% confidence intervals [95% CI].

Method	Version	Performance Index	Detected Walking Time (s)	Reference Walking Time (s)	Specificity	Accuracy	Recall	Precision	Absolute Relative Duration Error (%)	ICC
Kheirkhahan	wrist_improved	0.76	892.53 [681.32, 1103.74]	1028.44 [809.28, 1247.60]	0.96 [0.95, 0.98]	0.92 [0.90, 0.94]	0.55 [0.45, 0.64]	0.63 [0.53, 0.73]	35 [23, 47]	0.64 [0.36, 0.81]
Ionescu	wrist	0.75	925.50 [693.32, 1157.68]	1028.54 [809.37, 1247.72]	0.96 [0.95, 0.98]	0.92 [0.90, 0.94]	0.55 [0.46, 0.65]	0.63 [0.52, 0.74]	41 [27, 55]	0.60 [0.31, 0.79]
	wrist_adaptive	0.73	1043.35 [904.00, 1182.69]	1028.55 [809.37, 1247.74]	0.95 [0.94, 0.96]	0.91 [0.89, 0.93]	0.64 [0.54, 0.73]	0.59 [0.48, 0.69]	53 [27, 79]	0.47 [0.12, 0.71]
Keren	improved	0.69	579.21 [424.14, 734.29]	1028.94 [809.69, 1248.20]	0.98 [0.97, 0.98]	0.91 [0.89, 0.93]	0.36 [0.28, 0.45]	0.62 [0.51, 0.73]	44 [33, 54]	0.41 [−0.05, 0.71]
	adaptive	0.69	603.82 [445.95, 761.69]	1028.93 [809.68, 1248.19]	0.98 [0.97, 0.98]	0.91 [0.89, 0.93]	0.37 [0.28, 0.46]	0.60 [0.49, 0.70]	43 [33, 53]	0.43 [−0.03, 0.72]
Hickey	wrist_improved	0.65	1076.48 [817.62, 1335.34]	1028.74 [809.54, 1247.95]	0.92 [0.90, 0.95]	0.87 [0.84, 0.90]	0.43 [0.35, 0.50]	0.44 [0.35, 0.54]	65 [32, 99]	0.46 [0.11, 0.71]
MacLean	wrist	0.65	819.60 [631.30, 1007.90]	1028.62 [809.43, 1247.80]	0.94 [0.93, 0.96]	0.88 [0.85, 0.90]	0.31 [0.23, 0.39]	0.38 [0.29, 0.46]	31 [20, 42]	0.43 [0.09, 0.68]
Iluz	wrist	0.60	1208.85 [881.06, 1536.65]	1028.54 [809.36, 1247.72]	0.90 [0.87, 0.93]	0.85 [0.82, 0.88]	0.41 [0.32, 0.51]	0.38 [0.30, 0.46]	95 [49, 142]	0.37 [0.01, 0.65]

**Table 4:** Initial contact detection algorithm performance metrics ranked by performance index, with all metrics and error measures reported as mean values with 95% confidence intervals [95% CI].

Method	Version	Performance Index	Recall	Precision	Absolute Timing Error (s)	Relative Timing Error (%)
ShinIC	wrist	0.85	0.77 [0.72, 0.82]	0.82 [0.77, 0.87]	0.09 [0.08, 0.09]	12 [11, 13]
McCamleyIC	wrist	0.84	0.82 [0.79, 0.86]	0.77 [0.73, 0.80]	0.12 [0.11, 0.13]	16 [15, 18]
ZijlstraIC	wrist	0.83	0.78 [0.74, 0.82]	0.77 [0.73, 0.81]	0.12 [0.11, 0.13]	16 [15, 18]
DucharmeIC	wrist	0.82	0.76 [0.69, 0.83]	0.77 [0.74, 0.81]	0.12 [0.11, 0.13]	16 [15, 18]
GuIC	adaptive	0.82	0.69 [0.61, 0.77]	0.79 [0.76, 0.82]	0.10 [0.09, 0.10]	14 [13, 15]
HKLeeIC	wrist	0.82	0.77 [0.73, 0.81]	0.79 [0.76, 0.82]	0.13 [0.13, 0.14]	19 [17, 20]
GuIC	improved	0.82	0.67 [0.59, 0.74]	0.82 [0.78, 0.85]	0.10 [0.10, 0.11]	14 [13, 15]
PhamIC	wrist	0.82	0.77 [0.72, 0.82]	0.74 [0.70, 0.78]	0.12 [0.11, 0.13]	16 [15, 18]
Micó-Amigo	wrist	0.78	0.69 [0.64, 0.74]	0.65 [0.60, 0.70]	0.11 [0.10, 0.11]	15 [14, 16]

**Table 5:** Stride length estimation algorithm performance metrics ranked by performance index, with all metrics and error measures reported as mean values with 95% confidence intervals [95% CI].

Method	Version	Performance Index	Detected Stride Length (m)	Reference Stride Length (m)	Absolute Error (m)	Relative Error (%)	ICC
WeinbergSL	wrist	0.72	0.78 [0.73, 0.82]	0.76 [0.69, 0.84]	0.19 [0.16, 0.21]	31 [25, 37]	0.63 [0.34, 0.81]
	wrist_adaptive	0.71	0.77 [0.73, 0.82]	0.76 [0.69, 0.84]	0.19 [0.16, 0.22]	31 [24, 38]	0.62 [0.33, 0.81]
BylemansSL	wrist_adaptive_foot	0.67	0.77 [0.73, 0.82]	0.76 [0.69, 0.84]	0.20 [0.18, 0.22]	32 [27, 38]	0.54 [0.21, 0.76]
WeinbergSL	wrist_adaptive_foot	0.66	0.79 [0.76, 0.82]	0.76 [0.69, 0.84]	0.20 [0.18, 0.22]	35 [28, 42]	0.53 [0.20, 0.75]
	wrist_foot	0.66	0.79 [0.76, 0.82]	0.76 [0.69, 0.84]	0.20 [0.18, 0.22]	35 [28, 42]	0.53 [0.20, 0.75]
BylemansSL	wrist_foot	0.62	0.78 [0.75, 0.81]	0.76 [0.69, 0.84]	0.20 [0.18, 0.22]	35 [28, 42]	0.44 [0.08, 0.70]
	wrist	0.61	0.76 [0.71, 0.80]	0.76 [0.69, 0.84]	0.22 [0.19, 0.24]	34 [28, 40]	0.41 [0.05, 0.68]
KimSL	wrist_adaptive_foot	0.56	0.74 [0.72, 0.76]	0.76 [0.69, 0.84]	0.22 [0.19, 0.24]	35 [27, 44]	0.31 [−0.06, 0.61]
	wrist_foot	0.55	0.73 [0.71, 0.75]	0.76 [0.69, 0.84]	0.22 [0.19, 0.24]	35 [27, 43]	0.39 [0.04, 0.65]
BylemansSL	wrist_adaptive	0.55	0.65 [0.56, 0.73]	0.76 [0.69, 0.84]	0.33 [0.29, 0.37]	47 [40, 54]	0.30 [−0.07, 0.60]
KimSL	wrist_adaptive	0.44	0.73 [0.72, 0.74]	0.76 [0.69, 0.84]	0.23 [0.20, 0.26]	37 [28, 46]	0.06 [−0.32, 0.42]
	wrist	0.42	0.70 [0.70, 0.71]	0.76 [0.69, 0.84]	0.23 [0.20, 0.26]	37 [28, 45]	0.01 [−0.33, 0.36]

## Data Availability

The Python code developed for this study, including all novel algorithm implementations and parameter settings, is openly accessible at [https://github.com/DMegaritis/multimobility_wrist] and archived on Zenodo with DOI [version 1.0.0; https://doi.org/10.5281/zenodo.16926413] [18]. The complete dataset from the Mobilise-D Technical Validation study is available on Zenodo: https://zenodo.org/records/15861907.

## Supplementary Materials

The following supporting information can be downloaded at: https://www.mdpi.com/article/10.3390/bioengineering12101108/s1, Online Supplement.

## Abbreviations

The following abbreviations are used in this manuscript:

DMO	Digital Mobility Outcome
IMU	Inertial Measurement Unit
TVS	Technical Validation Study
MLTC	Multiple Long-Term Conditions
GSD	Gait Sequence Detection
IC/ICD	Initial Contact/Initial Contact Detection
SL	Stride Length Estimation
PFF	Proximal Femur Fracture
COPD	Chronic Obstructive Pulmonary Disease
PD	Parkinson’s Disease
MS	Multiple Sclerosis
CHF	Chronic Heart Failure
RMS	Root Mean Square
μ	Mean of the acceleration signal between two consecutive ICs
Δt	Time difference between two consecutive ICs
TP	True Positive
FP	False Positive
TN	True Negative
FN	False Negative
R/L	Right/Left (foot)
MAE	Mean Absolute Error
RMSE	Root Mean Squared Error
CWT	Continuous Wavelet Transform
SD	Standard Deviation
AI/ML	Artificial Intelligence/Machine Learning
