# Supplementary material for: Real-World Wrist-Derived Digital Mobility Outcomes in People with Multiple Long-Term Conditions: A Comparison of Algorithms
Source: Bioengineering (Basel). 2025 Oct 15;12(10):1108. doi: 10.3390/bioengineering12101108 (PMC12561645; doi:10.3390/bioengineering12101108)
Supplement: Supplementary file 1 [file bioengineering-12-01108-s001.zip › bioengineering-3871133-supplementary.pdf]

Supplementary Material to:

Real-world Wrist-derived Digital Mobility Outcomes in People with Multiple Long-term Conditions: A Comparison of Algorithms

Dimitrios Megaritis, Lisa Alcock, Kirsty Scott, Hugo Hiden, Andrea Cereatti, Ioannis Vogiatzis, and Silvia Del Din

Table of Contents

Methods ... ***2***

Performance metrics ... **2**

Table S1. Predefined performance metrics thresholds. ... **2**

Performance indices ... **3**

Table S2. Free-living - GSD performance metrics weights ... **3**

Table S3. ICD performance metrics weights ... **3**

Table S4. SL performance metrics weights ... **3**

Results ... ***4***

Figure S1. Bland–Altman plots illustrating the agreement and potential bias between sensor-derived and reference stride length measurements for the Bylemans algorithm. Each point is derived from a single walking bout, plotted as the difference between methods against their mean. Distributions of the stride length differences and means are shown along the top and right margins, respectively. Differences were calculated as wearable – reference, such that positive values indicate overestimation by the wearable and negative values indicate underestimation. Each point is colour codes based on the number of co-occurring long-term conditions. ... **4**

Figure S2. Bland–Altman plots illustrating the agreement and potential bias between sensor-derived and reference stride length measurements for the Kim algorithm. Each point is derived from a single walking bout, plotted as the difference between methods against their mean. Distributions of the stride length differences and means are shown along the top and right margins, respectively. Differences were calculated as wearable – reference, such that positive values indicate overestimation by the wearable and negative values indicate underestimation. Each point is colour codes based on the number of co-occurring long-term conditions. ... **5**

Figure S3. Plot of relative stride length error versus walking speed for the Bylemans algorithm. Each point reflects the relative error and corresponding walking speed calculated from a single walking bout. The figure illustrates how the wearable system's measurement error varies with walking speed. The fitted curve represents an exponential decay, $\epsilon = a\, e^{-b\,x} + c$, derived via non-linear least squares (parameters a, b, and c). The R^2 value indicates the goodness-of-fit between the data points and the fitted curve. Each point is colour codes based on the number of co-occurring long-term conditions. ... **6**

Figure S4. Plot of relative stride length error versus walking bout duration for the Bylemans algorithm. Each point reflects the relative error and corresponding walking bout duration calculated from a single walking bout. The figure illustrates how the wearable system's measurement error varies with walking bout duration. The fitted curve represents an exponential decay, $\epsilon = a\, e^{-b\,x} + c$, derived via non-linear least squares (parameters a, b, and c). The R^2 value indicates the goodness-of-fit between the data points and the fitted curve. Each point is colour codes based on the number of co-occurring long-term conditions. ... **7**

Figure S5. Comparison of performance index between original algorithm versions and the novel, improved, or fine-tuned versions developed in this study. ... **8**

Methods

Performance metrics

$$Accuracy = \frac{TP + TN}{TN + TP + FP + FN}$$

$$Recall\ (Sensitivity) = \frac{TP}{TP + FN}$$

$$Specificity = \frac{TN}{TN + FP}$$

$$Precision\ (Positive\ Predictive\ Value) = \frac{TP}{TP + FP}$$

where TP: True Positive; TN: True Negative; FP: False Positive; FN: False Negative

Table S1. Predefined performance metrics thresholds.

Metric	Poor	Acceptable	Good	Excellent
**GSD**				
Specificity	< 0.90	0.90–0.92	0.93–0.95	≥ 0.95
Accuracy	< 0.85	0.85–0.90	0.91–0.95	≥ 0.95
Recall (Sensitivity)	< 0.40	0.40–0.50	0.51–0.60	≥ 0.60
Precision (PPV)	< 0.50	0.50–0.60	0.61–0.80	≥ 0.80
Relative Duration Error (%)	> 50%	41–50%	30–40%	< 30%
ICC	< 0.50	0.50–0.60	0.61–0.70	> 0.70
**ICD**				
Recall (Sensitivity)	< 0.60	0.60–0.70	0.71–0.80	≥ 0.80
Precision (PPV)	< 0.70	0.70–0.80	0.81–0.89	≥ 0.90
Absolute Timing Error (s)	> 0.20 s	0.20–0.15 s	0.14–0.1 s	< 0.10 s
Relative Timing Error (%)	> 20%	20–16%	15–10%	< 10%
**SL**				
Absolute Error (m)	> 0.30 m	0.30–0.26 m	0.25–0.20 m	< 0.20 m
Relative Error (%)	> 40%	40–36%	35–30%	< 30%
ICC	< 0.40	0.40–0.50	0.51–0.60	> 0.60

Performance indices

Table S2. Free-living - GSD performance metrics weights

Metric	Classification	Weight
Absolute relative duration error	cost	0.185
ICC(2,1)	benefit	0.155
Specificity	benefit	0.203
Accuracy	benefit	0.185
Recall (Sensitivity)	benefit	0.142
Precision (Positive Predictive Value)	benefit	0.130

Table S3. ICD performance metrics weights

Metric	Classification	Weight
Recall (Sensitivity)	benefit	0.237
Precision (Positive Predictive Value)	benefit	0.229
Absolute error (s)	cost	0.267
Relative error	cost	0.267

Table S4. SL performance metrics weights

Metric	Classification	Weight
Absolute error (m)	cost	0.277
Relative error	cost	0.277
ICC(2,1)	benefit	0.446

Results

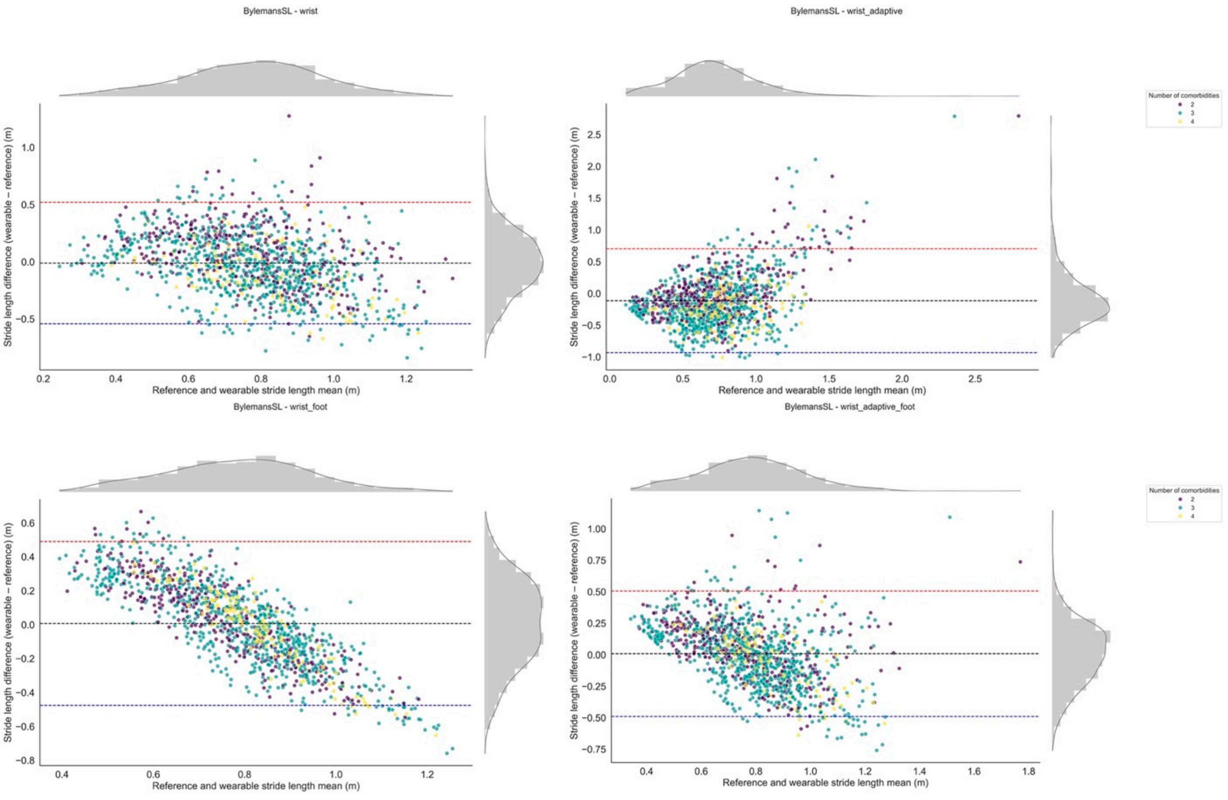

Figure S1. Bland–Altman plots illustrating the agreement and potential bias between sensor-derived and reference stride length measurements for the Bylemans algorithm. Each point is derived from a single walking bout, plotted as the difference between methods against their mean. Distributions of the stride length differences and means are shown along the top and right margins, respectively. Differences were calculated as wearable – reference, such that positive values indicate overestimation by the wearable and negative values indicate underestimation. Each point is colour codes based on the number of co-occurring long-term conditions.

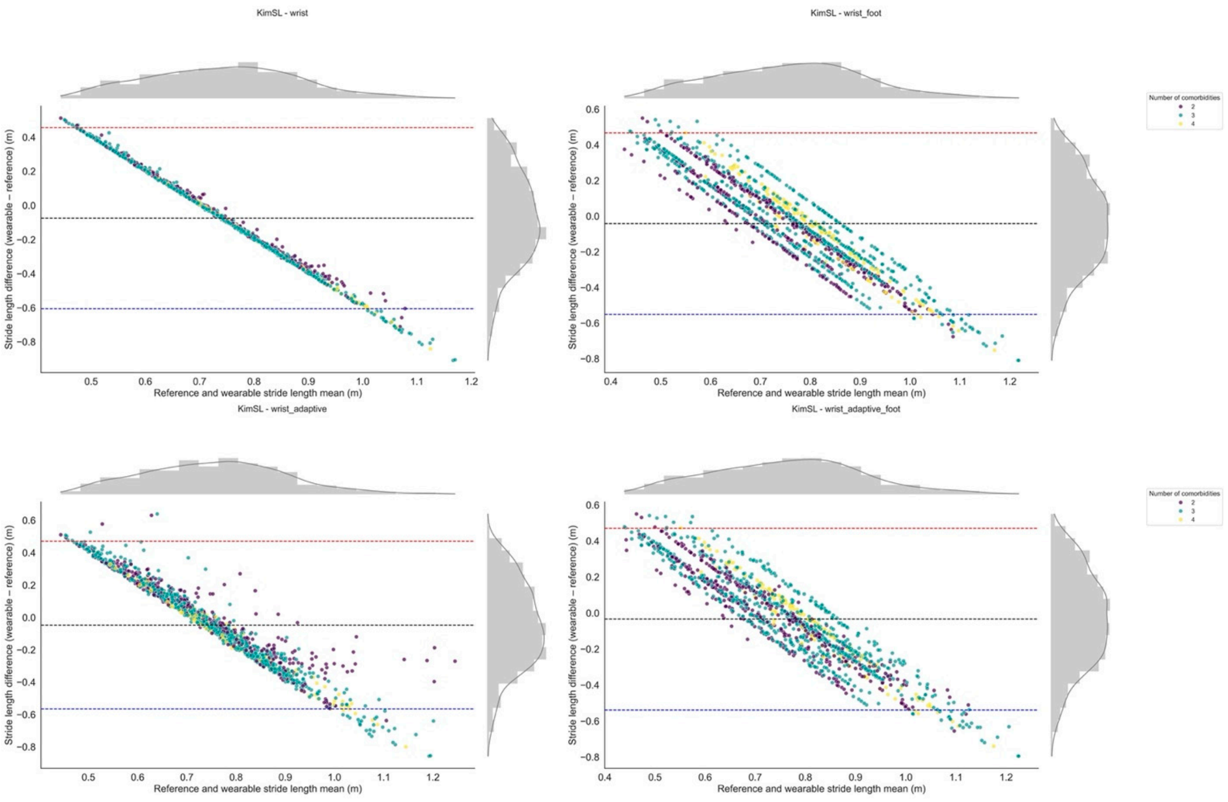

Figure S2. Bland–Altman plots illustrating the agreement and potential bias between sensor-derived and reference stride length measurements for the Kim algorithm. Each point is derived from a single walking bout, plotted as the difference between methods against their mean. Distributions of the stride length differences and means are shown along the top and right margins, respectively. Differences were calculated as wearable – reference, such that positive values indicate overestimation by the wearable and negative values indicate underestimation. Each point is colour codes based on the number of co-occurring long-term conditions.

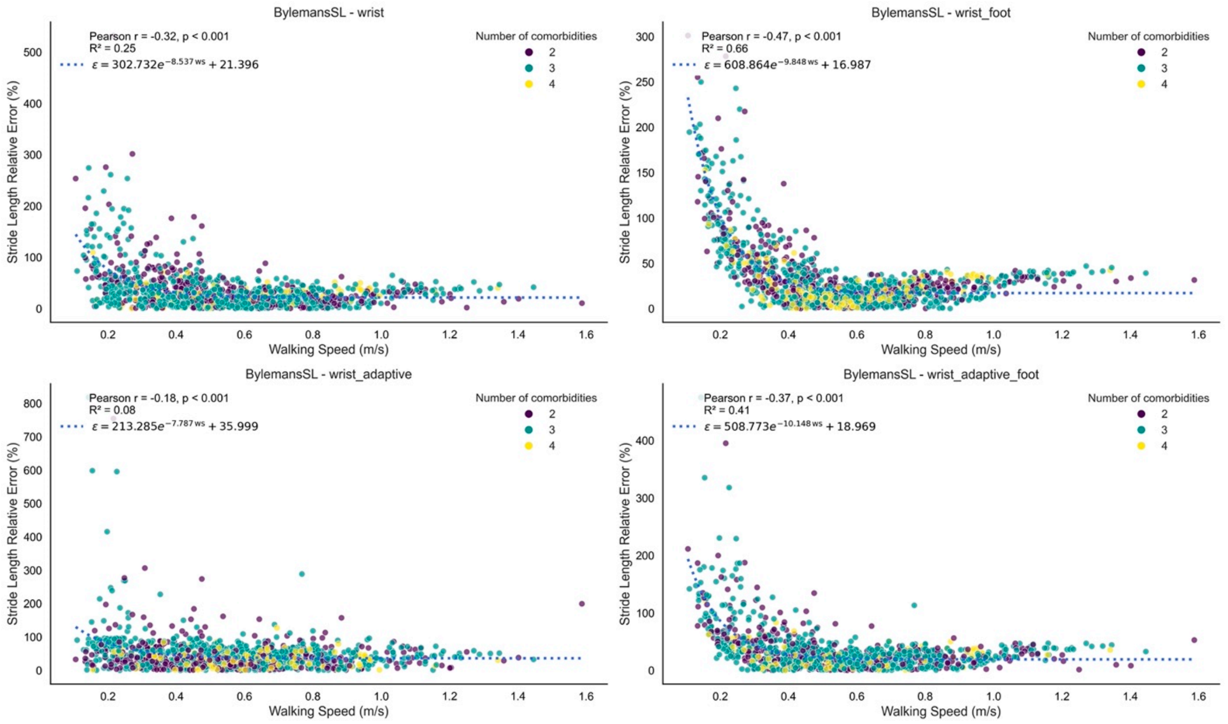

Figure S3. Plot of relative stride length error versus walking speed for the Bylemans algorithm. Each point reflects the relative error and corresponding walking speed calculated from a single walking bout. The figure illustrates how the wearable system's measurement error varies with walking speed. The fitted curve represents an exponential decay, $\epsilon = a\ e^{-\ b\ x} + c$, derived via non-linear least squares (parameters a, b, and c). The R^2 value indicates the goodness-of-fit between the data points and the fitted curve. Each point is colour codes based on the number of co-occurring long-term conditions.

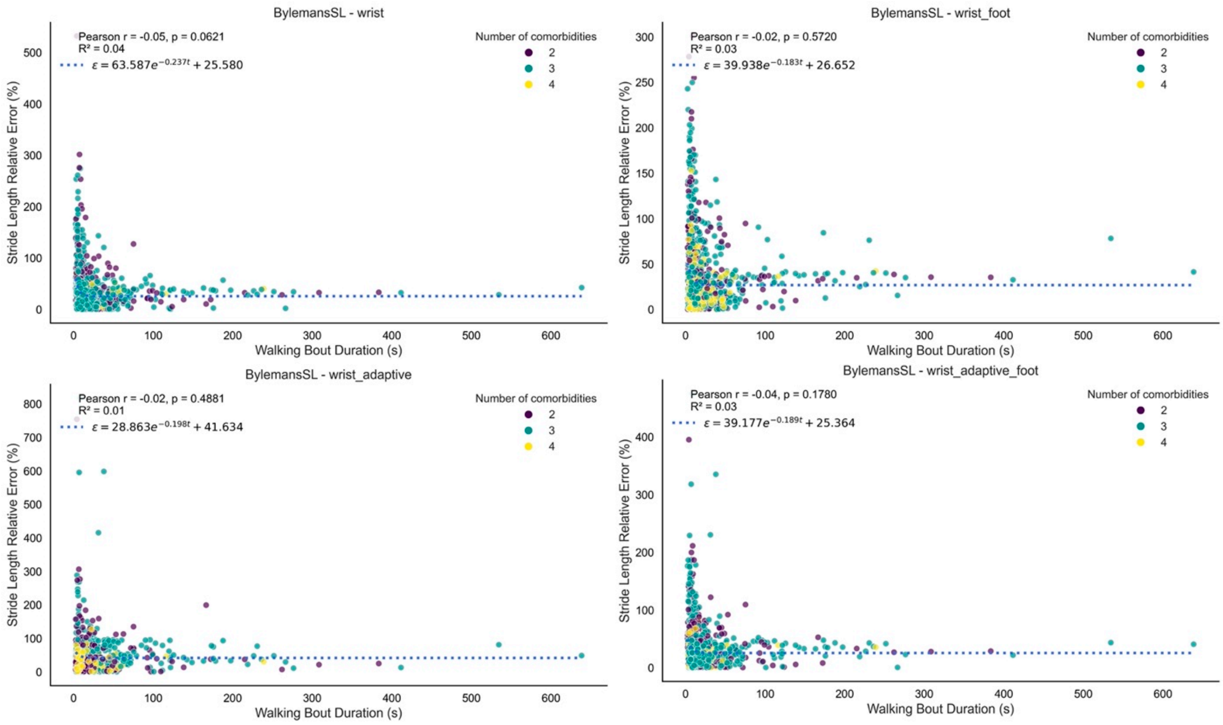

Figure S4. Plot of relative stride length error versus walking bout duration for the Bylemans algorithm. Each point reflects the relative error and corresponding walking bout duration calculated from a single walking bout. The figure illustrates how the wearable system's measurement error varies with walking bout duration. The fitted curve represents an exponential decay, $\epsilon = a\, e^{-\,b\,x} + c$, derived via non-linear least squares (parameters a, b, and c). The R^2 value indicates the goodness-of-fit between the data points and the fitted curve. Each point is colour codes based on the number of co-occurring long-term conditions.

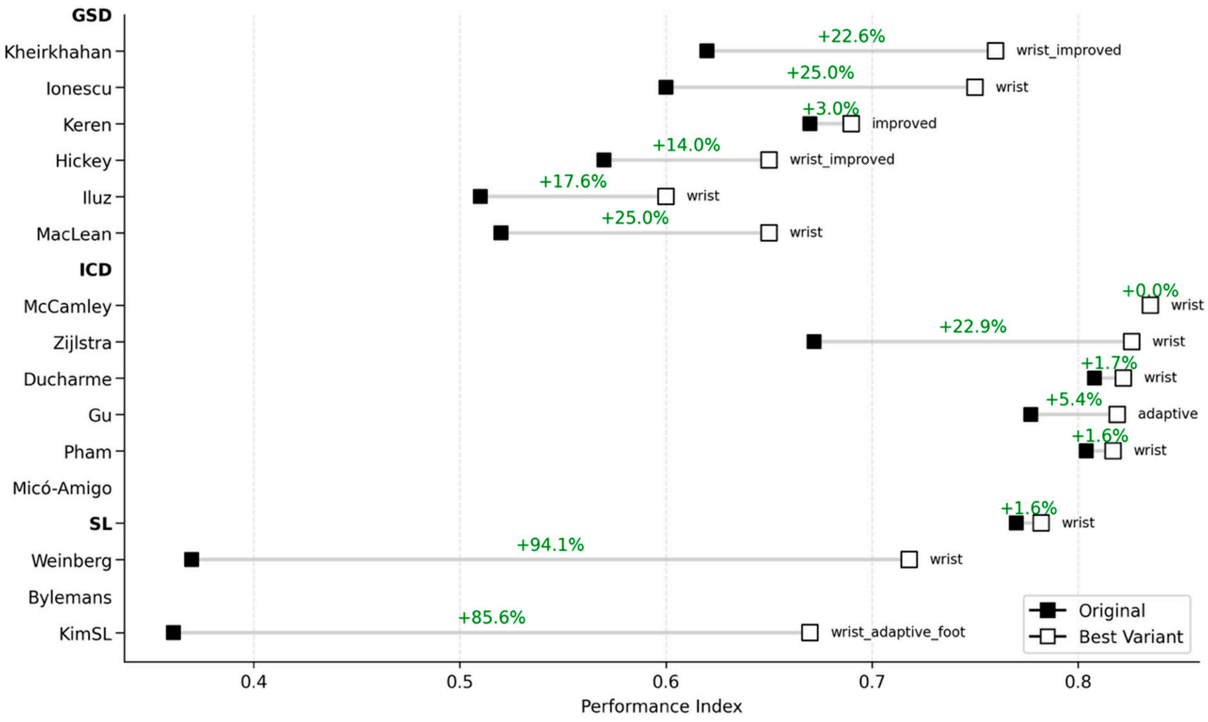

Figure S5. Comparison of performance index between original algorithm versions and the novel, improved, or fine-tuned versions developed in this study.
